# Association between residential environment and emotional wellbeing among older adults in China: the mediating effect of health lifestyle

**DOI:** 10.3389/fpubh.2024.1338079

**Published:** 2024-04-18

**Authors:** Zhu Huijie, Jiang Haojun, Zhu Zhiping, Yao Zhaoyu

**Affiliations:** ^1^College of Humanities and Social Development, Nanjing Agricultural University, Nanjing, China; ^2^Center for Social Research, Nanjing Agricultural University, Nanjing, China; ^3^Jin Shanbao Institute for Agricultural and Rural Development Research Institute, Nanjing, China; ^4^Tourism and Social Management College, Nanjing Xiaozhuang University, Nanjing, China

**Keywords:** emotional wellbeing, residential environment, older adults, mediating effect, health lifestyle, mental health disparity

## Abstract

**Introduction:**

The association between the residential environment and emotional wellbeing (EWB) in older adults has received extensive attention from gerontologists, especially during the COVID-19 pandemic; however, the mediating mechanism of how residential environment affects emotional wellbeing has not been fully explored. This study examined the effects of the residential environment on EWB and the mediating role of health lifestyle.

**Methods:**

This study analyzed the survey data of 493 rural and 515 urban older adults from 2021 Chinese General Social Survey. General linear regression and structural equation models were used to examine the effects of residential environment and health lifestyle.

**Results:**

Urban participants exhibited clear advantages in EWB, residential environment, and physical activity. Residential environment significantly affected the EWB of older adults, and health lifestyle played a mediating role in this relationship. The residential environment and health lifestyle did not significantly affect EWB in rural participants.

**Discussion:**

This study revealed differences in the effects of health lifestyles and residential environments on EWB among older adults in rural and urban settings in China. This study provided empirical evidence of mental health disparities between older rural and urban Chinese residents.

## 1 Introduction

With the deepening of aging globally, the health of the older population has received widespread attention. Population aged 65 and older accounts for 10% of the total population in 2023. Although life expectancy has generally increased, mental health problems among the older adults are becoming increasingly apparent. Researches have shown that, one in three individuals aged 65–84 years has experienced mental disorders within the past year, and one in four currently has a mental disorder (1). Emotional wellbeing (EWB) is one of the indicators measures a person’s mental health status, refers to the emotional quality of an individual’s everyday experience (2). EWB is related to a positive balance between pleasant and unpleasant affect and a cognitive appraisal of satisfaction with life in general (3). Emotional wellbeing can protect individuals against physical declines in old age (4). High levels of EWB have been associated with physical health, healthy aging, and longevity (5).

EWB has been examined from two perspectives: as a psychological aspect of wellbeing (6) and as a public health target (7, 8). As a psychological aspect of wellbeing, EWB is considered an indicator of an individual’s mental health, typically encompassing both positive and negative emotions (2). As a public health target, EWB is regarded as an indicator of health-related quality of life (9). EWB is closely related to the general health or wellbeing of individuals or populations (10, 11). Describing the general level of EWB among older population and analyzing its influencing factors is a meaningful public health issue, especially in the context of worldwide aging. Among the factors that affect the EWB of older adults, we focused on examining the effect of residential environment. After older adults leave their jobs, they mainly live in the community residents, which plays an important role in their later life.

### 1.1 EWB of older adults and emotional aging

EWB run exists throughout one’s lifespan (12) and is closely related to age (13). Research has focused on the EWB of children (14) and students (15). With the acceleration of global aging, EWB has become an important issue concerning health in old age. Emotional aging is an inevitable process experienced by older adults. Studies have examined the developmental logic of EWB across the lifespan (16) which have reached two opposing conclusions (17). On one hand, the emotional health of the older adults is on a continuous decline trend. Dynamic Integration Theory (DIT) argue that the efficiency of emotion systems is a dynamic interplay and flexible trade-off between contextual variables and individual characteristics (18). Limitations or poor regulation strategies foster compensatory processes that compromise integration later in life (19). Older adults are at high risk for mental illnesses, such as loneliness (20) and depression (21). Depressive symptoms predict increased social and emotional loneliness in older adults (22), resulting in poor mental health (23).

On the other hand, older people benefit from emotional aging (12, 24). Older adults exhibited a better EWB than young and middle-aged people (25, 26). The Socioemotional Selectivity Theory (SST) posits those changes in time perception cause changes in motivation for social interaction among older adults, thereby improving the quality of social networks (27). Charles (28) argued that aging should be considered an adaptation that sheds light on resilience, wellbeing, and emotional stress in adulthood.

Although there is no consensus on the level of emotional health of the older adults throughout their life span, the emotional health of the older population and its influencing factors have received attention from researchers. Research on the differences in the EWB of older adults has investigated factors affecting emotional health, including individual behavior, social resources, and family factors. Individual behavior includes exercise (29), social media use (30), volunteerism (31) and discrimination based on age (32). Social resources include social support (33), social cohesion (34), personal and mobility resources (35), and long-term care insurance (36). Family factors include intimate relationship strain (37), intergenerational family relationships (38), and family support (39). For older adults, individual behavior, social resources, and family factors exist in their residential environments; however, these factors relate more to behavioral consequences and action resources.

### 1.2 Residential environment and EWB

Community-dwelling older adults are mainly active in the community after retirement (40). The effect of community environment on the health of older adults is lasting and stable. Community is an important factor in analyses of the EWB of older adults. Community psychology provides a disciplinary basis for analyzing the relationship between the residential environment and EWB. From the perspective of community psychology, community residents are not atomized individuals but, rather, community participants and practitioners (41). Health is affected by the community-built environment (42), community participation (43), and the neighborhood environment (44). Community participation enhances the subjective wellbeing of older adults by increasing their sense of community (45). Social contact has a positive effect on the health of older people who live alone, especially in-person contact with non-family members (46).

Research has examined the mechanisms by which the community environment affects EWB. The verified mediating variables included social cohesion, neighborhood security (47), social support (48), and sense of community (49). These mediating variables play a role at the psychological or behavioral level, focusing on the impact path of the residential environment on EWB from a psychological perspective.

From a public health perspective, EWB is both a right to health and a health outcome. EWB is related to the health equity of the older population. Residential environment is related to an individual’s social status and resources and is affected by the social structure. EWB, as a health outcome, is influenced by residential environment. Focusing on the mediating mechanism of the EWB of residential environment among older adults reveals the social logic of health inequality. Therefore, exploring how inequality in residential environments translates into disparity in EWB among older adults in China is an important public health issue. The current literature focuses on the effect of socioeconomic factors on EWB, but insufficient attention is paid to residential environments. There are few studies on the mediating mechanism of EWB in older adults affected by living environment. We used data from CGSS 2021 to focus on the impact of residential environment on EWB among older adults in urban and rural areas, with a focus on the mediating role of health lifestyles. The study revealed the residential environment factors and mechanisms that affect EWB of older adults. The conclusion shows that EWB of the old adults is an emerging public health issue in Mainland China. It is necessary to intervene in the inequality in EWB from the perspective of community environment and health lifestyle.

## 2 Theoretical model and hypotheses

### 2.1 Theoretical model

**Figure 1:**
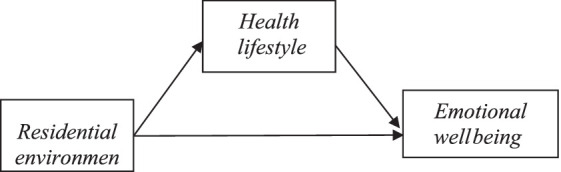
Theoretical model.

The socio-ecological model provides theoretical support for understanding the positive role of communities in health. McLeroy et al. (50) introduced the social-ecological model into health research, noting that behavior is determined by intrapersonal factors, interpersonal processes, primary groups, institutional factors, community factors, and public policies. The socio-ecological model is often used to analyze the influencing factors of health-related factors, such as eating behaviors (51), physical activity (52), and mental wellbeing (53). The socio-ecological model differs from traditional psychological models in that it explicitly combines direct external influences with peripheral factors, making the socio-ecological model popular in the public health realm (54). Communities play a key role in the social system of community-dwelling older adults. Community is the main space for the social activities of older people and is closely related to their life quality (55). In Mainland China, older people in rural and urban areas tend to remain in their communities rather than moving to nursing homes (56). Therefore, residential environments may play an important role in the wellbeing of older adults in both urban and rural areas in China (Figure 1).

The health lifestyle theory reveals the microscopic mechanism of health disparity from the perspective of social stratification and introduces structure (57) to understand the mediating mechanism of residential environment’s effect on the EWB of older adults. Health lifestyles are collective patterns of health-related behaviors based on choices among options available to people according to their life opportunities (58). Health lifestyles include smoking, drinking, sleep, physical activity, and other daily behaviors related to health (59). Health is the outcome of social shaping in which health lifestyle plays a key role (60). According to the health lifestyle theory, health lifestyle is an important medium shaping individual health (61), and residential environment sets the social and cultural standards (62). Residential environment is an external feature of social class (63), and health lifestyles act as an intermediary variable affecting EWB (64). Therefore, lifestyle positively affects individual health.

### 2.2 Hypotheses

Given that the association between residential environment and EWB among the older adults has not been thoroughly explored, this study attempts to construct hypothesizes on the association between residential environment and EWB, and tests the mediating effect of health lifestyles.

According to the disengagement theory, most older adults return from public engagement to community life toward the end of their careers (65). They live in a community environment long term and interact closely with their neighbors; therefore, the residential environment is a key factor affecting their quality of life. Residential environment exerts a strong impact on the subjective wellbeing of older adults (66, 67). Variables of residential environment conclude internal and external conditions of the home (68), Interior living environments (69), out-of-home activities (70). Based on the analysis framework set earlier and the results of existing literatures, we have set the hypothesis 1.

*H*1: *Residential environment is associated with older adults’ EWB.*

The health lifestyle theory provides theoretical support for the mediating effect of health lifestyle on the relationship between residential environment and EWB. Studies have suggested that health lifestyles are closely related to EWB of older adults. According to the theory of healthy lifestyle, a person’s health situation is outcome of their health lifestyle (60). The health status of older adults living in the community is influenced by community activities (71), while the quality of community life is influenced by the residential environment (42). Healthy lifestyle variables generally include physical activity and sleep quality. Physical activity promotes psychological wellbeing in older adults (72–74), and higher sleep quality indicates better EWB and quality of life (75, 76). Thus, health lifestyles significantly affect EWB.

Health lifestyles are closely related to residential environments. Based on the socio-ecological model, the residential environment is an objective aspect of health lifestyle. According to the health lifestyle theory, the residential environment is a community factor that forms an individual’s health lifestyle. Moreover, environmental factors influence physical activity (77). Studies have provided empirical evidence for the association between the residential environment and health lifestyle variables, such as physical exercise (78) and sleep quality (79). Residential environment and health lifestyle affect EWB, and residential environment affects health lifestyle, providing empirical data to support the mediating effect of health lifestyle.

*H*2: *Health lifestyle plays a mediating role in the relationship between the residential environment and EWB.*

Hypotheses 1 and 2 address the association between the residential environment and EWB and the mediating effect of health lifestyle. Considering the particularity of the Chinese context, the study also examined the mediating effect of health lifestyle in urban and rural settings. Significant differences exist in the residential environment, health lifestyles, and wellbeing of urban and rural older adults (80, 81). Most rural adults work in agriculture when they are young and live in relatively traditional farming communities later in life (82). Most adults in urban areas work in non-farm jobs in factories or units and live in relatively modern urban communities when they are old (83). The mediating effects of health lifestyle may differ between urban and rural older adults. Therefore, we proposed the following hypothesis:

*H*3: The mediating effect of health lifestyle among older adults differs between urban and rural areas.

## 3 Methods

### 3.1 Data and sample

This study used secondary data from the Chinese General Social Survey (CGSS). The CGSS is part of the International Social Survey Programme (ISSP). Users can utilize these open-source data after obtaining permission (84). This study has been authorized to use the data in question. The 2021 Chinese General Social Survey was conducted using a multi-stage stratified probability proportionate-to-size (PPS) random sampling method. A total of 8,148 valid samples were recovered from 320 communities and 19 provinces. The target population for this study consisted of Chinese adults over the age of 60 years living in urban and rural areas. Because the mobility of Chinese older adults is low and this study takes residential environment as the key variable, the definition of older adults in urban and rural areas is mainly based on the living area where the survey is conducted. After removing samples with missing core variables, 1,008 valid samples were obtained (493 rural and 515 urban older adults) (Table 1).

**Table 1:** Characteristics of the total sample.

	Measurement	MIN	MAX	Mean	SD
Urban	Urban = 1, rural = 0	0	1	0.510	0.500
Age	The difference between year of survey minus year of birth	60	99	70.410	7.081
Male	Male = 1, female = 0	0	1	0.491	0.500
Marital status	Married = 1, not married = 0	0	1	0.701	0.458
Education	Years of schooling	0	19	6.732	4.424
Family economic	1 = well below the average; 5 = well above the average	1	5	2.550	0.808

### 3.2 Measurement

#### 3.2.1 Dependent variable

EWB was measured using a scale containing a set of questions regarding the emotional state of the respondents. Five questions were asked: (1) Are you unable to complete expected work or daily activities due to emotional problems? (2) Do you have emotional problems that distract you at work or during other daily activities? (3) In the past 4 weeks, to what extent have your physical health or emotional problems affected your social activities, such as visiting friends and relatives? (4) Do you feel calm? (5) Are you full of energy? Responses were rated on a five-point Likert scale (1 = never; 5 = always). Items in the scale relate to both negative and positive emotions. To facilitate the analysis, the negative emotion rating was reversed: the lower the frequency, the less frequently the individual experienced negative emotions. The Cronbach’s alpha coefficient of this scale was 0.787, indicating good reliability. To reduce the dimensions of the scale, we performed a factor analysis. The KMO score was 0.754, indicating that this scale was suitable for factor analysis. Factor analysis was performed, and the cumulative variance contribution rate was 55.376%, which was named the EWB factor. As the mean value of the extracted common factors was 0, it could not be used to compare differences in EWB among different populations. We converted the common factors into a percentage system (the converted factor value = (factor value + B) * A, A = 99/(MAX_factor_ − MIN_factor_), B = (1/A) − MIN_factor_), and the interval was between 0 and 99.

#### 3.2.2 Independent variables

Residential environments were measured using the respondents’ subjective evaluations. A four-item scale was designed as follows: (1) Where I live is suitable for physical exercise, such as jogging and walking; (2) There are many fresh vegetables and fruits to choose from where I live; (3) There are enough public facilities where I live; and (4) Where I live is safe. Responses were rated on a five-point Likert scale (1 = strongly disagree; 5 = strongly agree). The Cronbach’s alpha coefficient of the scale was 0.585, indicating acceptable reliability. The KMO score was 0.682. Factor analysis was performed, and the cumulative variance contribution rate was 46.039%. A common factor was extracted and named the residential environment factor.

Physical activity and sleep quality were used to measure health lifestyles. When operationalizing health lifestyles, this study did not select variables such as smoking, drinking, eating habits and sedentary behaviors, mainly for the following two reasons: on the one hand, there are significant regional and cultural differences in the eating habits in China, especially for Chinese older adults, which will to some extent offset the impact of living environment on emotional health; on the other hand, the dataset we used did not investigate variables related these behaviors, physical activity and sleep quality can to some extent reflect a person’s health lifestyle. Physical activity was measured using self-reported frequency of physical exercise. Respondents were asked the following question: In the past year, have you often participated in physical exercise in your leisure time? Responses were rated on a five-point Likert scale (1 = never; 5 = every day). Sleep quality was self-rated on a four-point Likert scale (1 = very poor; 4 = very good).

#### 3.2.3 Control variables

Sociodemographic variables, including age, gender, marital status, education, and family economic status, were used as control variables. Age was obtained by subtracting the year of survey from the year of birth. Gender was converted to a dummy variable representing men. Marital status was converted to having a spouse. Education was converted into the number of years of schooling. Family economic status was self-rated, with higher values indicating better family economic status. The control variables were not key variables, and their significance was not listed in the model. The regression model reported whether control variables were selected.

### 3.3 Statistical analyses

As this study aimed to explore the relationship between multiple variables, we used a structural equation model (SEM). SPSS 24.0 and AMOS were used for statistical analyses. SPSS was used to analyze the relationships between variables. The differences between urban and rural areas in these core variables were tested using a one-way analysis of variance (ANOVA). General linear regression (OLS) was used. In addition, AMOS was used to analyze SEMs.

## 4 Results

### 4.1 Residential environments and EWB of the older adults

Table 2 presents the key variables. Older adults in urban areas had better EWB, residential environments, and physical activity than those in rural areas. There was no significant difference in sleep quality between older adults in urban and those in rural areas.

**Table 2:** Key variables of urban and rural older adults.

	Urban		Rural		All		*F* (*p*)
	Mean	SD	Mean	SD	Mean	SD	
EWB	75.10	18.72	68.32	20.89	71.78	20.09	29.453 (0.000)
RE	70.34	19.01	64.63	18.55	67.56	18.98	23.305 (0.000)
PA	3.23	1.74	2.17	1.65	2.71	1.78	99.541 (0.000)
SQ	2.81	0.831	2.86	0.885	2.84	0.858	0.802 (0.371)

EWB, emotional wellbeing; RE, residential environment; PA, physical activities; SQ, sleep quality. The same below.

The correlations between EWB and related variables were tested to analyze the complex relationships among the key variables (Table 3). There was no significant correlation between the urban dummy variable and sleep quality, whereas the other variables exhibited significant correlations. These results were used for the regression and SEM analyses.

**Table 3:** Correlation analysis results for EWB and related variables.

	Urban	RE	PA	SQ	EWB
Urban	1				
RE	0.150^***^	1			
PA	0.300^***^	0.222^***^	1		
SQ	−0.028	0.149^***^	0.085^**^	1	
EWB	0.169^***^	0.192^***^	0.221^***^	0.313^***^	1

***p < 0.001, **p < 0.05, *p < 0.01.

Five linear regression models of EWB among older adults were established (Table 4). Model 1 included residential environment, physical activity, and sleep quality. Model 2 included the urban variable in addition to the Model 1 variables. Model 3 further added the control variables. In Models 4 and 5, the core and control variables were added as independent variables. Models 4 and 5 were urban and rural models, respectively. The adjusted coefficient of determination for Model 1 was 14.5%. The explanatory power of Model 3 was the highest at 18.1%.

**Table 4:** OLS models of the participants’ EWB.

	Model 1	Model 2	Model 3	Model 4-urban	Model 5-rural
RE	0.119 (0.032)^***^	0.106 (0.032)^**^	0.104 (0.031)^***^	0.128 (0.040)^**^	0.083 (0.049)
EB	1.953 (0.339)^***^	1.566 (0.350)^***^	1.210 (0.356)^***^	1.774 (0.448)^***^	0.549 (0.566)
SQ	6.588 (0.691)^***^	6.777 (0.688)^***^	6.369 (0.687)^***^	6.867 (0.914)^***^	5.959 (1.033)^***^
Urban		4.828 (1.226)^***^	4.691 (1.282)^***^		
Age			−0.193(0.086)^*^	−0.090(0.106)	−0.331(0.140)^*^
Male			3.220(1.244)^*^	1.735(1.621)	4.719(1.919)^*^
Marital status			1.540(1.335)	1.735(1.735)	1.283(2.062)
Education			0.349(0.152)	0.365(0.181)^*^	0.327(0.260)
Family economic			−0.349(0.040)^*^	−0.037(0.046)	−0.057(0.069)
(Constant)	39.797 (2.738)^***^	38.679 (2.734)^***^	49.790 (6.898)^***^	42.739 (8.834)^***^	62.978 (10.986)^***^
Adjusted *R*^2^	0.145	0.157	0.181	0.193	0.126

***p < 0.001, **p < 0.05, *p < 0.01.

In Model 1, residential environment, physical activity, and sleep quality showed significant positive relationships. This indicated that better residential environment, higher frequency of physical activity, and higher sleep quality were associated with better EWB. All variables in Model 2 were significant. The urban dummy variable was significant, indicating that EWB was higher in urban than rural areas. In Model 3, the significance of the independent variables did not change after introducing the control variables, indicating a stable and significant correlation between EWB and these independent variables. Thus, Hypothesis 1 was supported.

According to Model 4, residential environment, physical activity, and sleep quality positively affected EWB in urban areas. According to Model 5, only sleep quality positively affected EWB in rural areas. The explanatory power of independent variables differed in the urban and rural models; therefore, urban and rural samples were analyzed separately.

### 4.2 Mediating effect of health lifestyle

To test the mediating effect of health lifestyles on residential environments and EWB, we conducted an SEM analysis. The chi-square value of the default model was 2.963 (*p* = 0.085), the root mean square error of approximation (RMSEA) was 0.044, the comparative fit index (CFI) was 0.992, and the Tucker-Lewis index (TLI) was 0.949. These indicators showed that the mediation model met the requirements of the SEM, and the mediation effect model was valid and effective.

**Figure 2:**
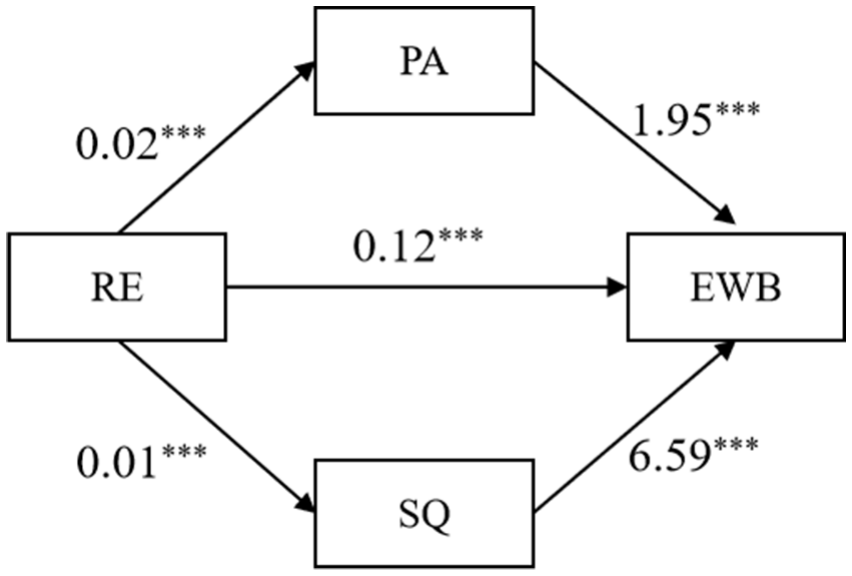
The mechanism of residential environments affecting EWB.

Figure 2 shows the mechanism through which residential environments affected EWB. Physical activity and sleep quality mediated the relationship between residential environment and EWB. The model revealed a mediating effect of health lifestyles.

The variable effects of the SEM are presented in Table 5. Physical activity and sleep quality had mediating effects, supporting Hypothesis 2.

**Table 5:** Variable effects in the SEM model.

	Estimate	SE	CR	*p*-value	Total effect	Direct effect	Indirect effect
SQ ← RE	0.007	0.001	4.797	<0.001	0.149	0.149	0.000
PA ← RE	0.021	0.003	7.220	<0.001	0.222	0.222	0.000
EWB ← RE	0.119	0.032	3.719	<0.001	0.193	0.112	0.081
EWB ← SQ	6.588	0.689	9.559	<0.001	0.282	0.282	0.000
EWB ← PA	1.953	0.338	5.781	<0.001	0.173	0.173	0.000

An SEM was used to analyze urban and rural samples. The variable effects are shown in Table 6. The urban model revealed five influence paths. In the rural model, the EWB ← PA and EWB ← RE paths were not significant, indicating that the residential environment did not affect EWB. Physical activity, which was a healthy lifestyle variable, had no significant effect on the EWB of rural older adults.

**Table 6:** Variable effects in urban and rural models.

	Urban model			Rural model		
	Standardized estimate	Total effect	Indirect effect	Standardized estimate	Total effect	Indirect effect
SQ ← RE	0.126^**^	0.126	0.000	0.185^***^	0.185	0.000
PA ← RE	0.192^***^	0.192	0.000	0.182^***^	0.182	0.000
EWB ← RE	0.132^***^	0.209	0.077	0.072	0.137	0.065
EWB ← SQ	0.318^***^	0.318	0.000	0.276^***^	0.276	0.000
EWB ← PA	0.192^***^	0.192	0.000	0.078	0.078	0.000

***p < 0.001, **p < 0.05.

In the urban model, health lifestyle variables play a partial mediating effect between Residential environments and EWB, while in the rural model, only sleep quality plays a complete mediating effect. By comparing the urban and the rural model, it was found that health lifestyle plays a mediating role, but the mechanism behind the difference among the urban and rural models needs further analysis.

## 5 Discussion

Our study highlights the mediating role of health lifestyle in the residential environment affecting EWB among older adults in China. This study found that residential environments have a positive impact on EWB among the Chinese older-adult population, which is consistent with the consensus of community psychology (85). Based on the health lifestyle theory, we raised this mechanism from the individual level to the hierarchical level. Health lifestyle does not refer to an individuals’ health lifestyle; it represents the collective lifestyle of a certain social stratum (57). Living conditions are a structural variable that can impair, maintain, or promote health (86). Socioeconomic status has the strongest effect on health (87, 88). This study found that for older adults with lower socioeconomic status, who tend to have low-quality residential environments and relatively unhealthy lifestyles, socioeconomic status negatively impacted EWB. This revealed the mechanism by which status affects older adults’ mental health. Numerous studies have focused on the health hazards caused by natural environmental hazards (89); however, few studies have examined the cumulative effects of the built (90) and humanistic environments (91) on mental health. The health lifestyle theory focuses more on lifestyles conducive to health promotion than environmental exposure to health hazards.

Our study confirmed that mental health disparities are widespread among the older population in China. The role of residential environment and health lifestyle in the formation of EWB disparity in the older population is significant, and prior exposure to housing disadvantage may impact mental health later in life (92). The living environment extends the living disadvantage to the community level beyond housing conditions. Macrosocial structure has a profound influence on mental health through community life as a mesoscopic path and daily life as a microcosmic path. Thus, promoting health equity in the relationship between class structure and individual EWB requires improving the residential environment and fostering good health lifestyles through public health policies implemented by the government. Old age is the later stage of life, and health disparities are the outcome of the accumulation of health behaviors, resources, and risks (93). Mental health disparities in old age are difficult to overcome using individual resources and require broader public health policies and social synergies. Health equity should include health throughout the lifecycle, and health equity in old age should not be ignored.

This study examined the urban–rural differences in the mediating effects of health lifestyles. Widespread mental health disparities exist between urban and rural areas in China (94). In China, urban and rural areas exhibit regional differences and are a key element of the social structure that affects health and healthcare (95, 96). Chinese adults over the age of 60 were affected by the reform and opening-up policy in their early adulthood. Older adults in urban areas live in more modern communities than rural older adults, and their lifestyle is becoming relatively healthier and more modern (80). The effects of residential environment and health lifestyle were observed only in modern urban communities, with no significant effect on EWB among older people in rural areas. As China continues to modernize, the effects of health lifestyles may emerge in rural areas.

The effects of the residential environment and health lifestyle on EWB differed between urban and rural areas. This effect exists among the urban older population but does not apply to the rural older population. We believe that the differences in the mediating effects of health lifestyles between urban and rural areas can be analyzed from both resource perspective and culture perspective. From the resource perspective, China have created a two-class society based on hukou status with sharp rural–urban distinctions in the public provision of health care and housing (97). the payment ability and residential environment quality of urban older adults are better than those of rural older adults (98). Most rural older adults generally have poor residential environments, which do not affect their EWB. Urban communities have developed public infrastructure, making urban communities generally liveable; however, rural communities are mainly composed of natural landscapes, and there is little difference in living environment. Older adults in urban communities showed advantages in terms of residential environment, health lifestyle, and EWB compared to those in rural communities. In addition, the heterogeneity of these variables in the urban older population was higher than that in the rural older population; therefore, older adults in rural communities exhibited disadvantages in terms of EWB and health lifestyle. From a cultural perspective, cities and rural areas are different cultural systems. In *Peasant Society and Culture: An Anthropological Approach to Civilization*, Redfield (99)proposes the great tradition represented by cities and the little tradition represented by the countryside. “The great tradition” implies the heterogeneity of personal characteristics and means that to pursuit a healthy lifestyle. In contrast, older adults in “the little tradition” have strong homogeneity and follow traditional and conventional lifestyles. Therefore, resources and culture are two possible explanatory paths. Further research is needed to verify these two explanatory pathways.

There are several limitations in this study. EWB has not been clarified (5). The study was based on the analysis of secondary data, and the operationalization and measurement of variables were limited. In addition, the study only analyzed community-dwelling older adults and did not include older people living in nursing homes. The reasons for the differences between urban and rural areas in the effects of residential environment and the mediating effects of health lifestyle also need to be further analyzed and verified.

## 6 Conclusion

This study examined the effects of the residential environment on EWB and the mediating role of health lifestyles among older adults in urban and rural communities in China. Our findings revealed that older adults in urban areas had better EWB, residential environment, and physical activity than those in rural areas. The effect of residential environment on EWB were demonstrated, and health lifestyle played an important mediating role. Residential environment and health lifestyle did not have a significant effect on EWB in rural older adults. This study provided empirical evidence of mental health disparities among older adults in rural and urban areas of China. The study reveals the important role of health lifestyle in translating inequality in residential environments into disparity in EWB. To counter the effects of such health inequalities, it is necessary to foster healthier lifestyles and improve the quality of the residential environment within communities.

## Data availability statement

The original contributions presented in the study are included in the article/supplementary material, further inquiries can be directed to the corresponding author.

## Author contributions

ZH: Conceptualization, Formal analysis, Funding acquisition, Methodology, Writing – original draft, Writing – review & editing. JH: Data curation, Methodology, Writing – review & editing. ZZ: Methodology, Visualization, Writing – review & editing. YZ: Conceptualization, Formal analysis, Project administration, Supervision, Validation, Writing – review & editing.
